# Structure-Based GC Investigation Sheds New Light on ITS2 Evolution in *Corydalis* Species

**DOI:** 10.3390/ijms24097716

**Published:** 2023-04-23

**Authors:** Qing Xian, Suyin Wang, Yanyan Liu, Shenglong Kan, Wei Zhang

**Affiliations:** 1 Marine College, Shandong University, Weihai 264209, China; xianqing2021@163.com (Q.X.);; 2 College of Plant Protection, Henan Agricultural University, Zhengzhou 450002, China; 3 Shenzhen Branch, Guangdong Laboratory of Lingnan Modern Agriculture, Genome Analysis Laboratory of the Ministry of Agriculture and Rural Affairs, Agricultural Genomics Institute at Shenzhen, Chinese Academy of Agricultural Sciences, Shenzhen 518120, China

**Keywords:** internal transcribed spacer 2, GC-biased evolution, GC content, secondary structure, concerted evolution

## Abstract

Guanine and cytosine (GC) content is a fundamental component of genetic diversity and essential for phylogenetic analyses. However, the GC content of the ribosomal internal transcribed spacer 2 (ITS2) remains unknown, despite the fact that ITS2 is a widely used phylogenetic marker. Here, the ITS2 was high-throughput sequenced from 29 *Corydalis* species, and their GC contents were comparatively investigated in the context of ITS2’s characteristic secondary structure and concerted evolution. Our results showed that the GC contents of ITS2 were 131% higher than those of their adjacent 5.8S regions, suggesting that ITS2 underwent GC-biased evolution. These GCs were distributed in a heterogeneous manner in the ITS2 secondary structure, with the paired regions being 130% larger than the unpaired regions, indicating that GC is chosen for thermodynamic stability. In addition, species with homogeneous ITS2 sequences were always GC-rich, supporting GC-biased gene conversion (gBGC), which occurred with ITS2’s concerted evolution. The RNA substitution model inferred also showed a GC preference among base pair transformations, which again supports gBGC. Overall, structurally based GC investigation reveals that ITS2 evolves under structural stability and gBGC selection, significantly increasing its GC content.

## 1. Introduction

The content of guanine and cytosine (GC) is an important feature of genomic composition and the material foundation for species diversity [1]. The average content of genomic GC ranges greatly from 20% to 60% in eukaryotes and 13% to 75% in bacteria [2,3], creating vast genome diversity in the tree of life. The GC content of the grass genome in higher plants is significantly higher than that of other angiosperms [4]. GC, in particular, distributes heterogeneously and forms a distinct bimodal pattern in Poaceae plants [5,6]. In some mammals and birds, GC distributes in a patchwork pattern, with 100-kb large GC-rich and GC-poor regions alternating along the genome to form a well-known isochore structure [7].

Although genomic GC variation has been investigated for over half a century, the mechanism of GC variation is still uncertain. Traditionally, three main hypotheses have been proposed to account for the genomic GC variation in distinct evolutionary scenarios, including selection, mutational biases, and GC-biased gene conversion (gBGC). Selection is typically applied to the GC content of coding sequences, with translational selection driving the codon usages of the highly expressed gene, resulting in higher GC contents if preferred codons primarily end in G or C [2,8,9]. The mutation hypothesis focuses on the mutation hotspots along the genomes that drive GC content [10], such as the methylation variable position, which tends to increase the mutation bias toward AT. The mutation direction is also determined by the availability of free nucleotides during DNA replication [11]. In recent years, the gBGC hypothesis has been increasingly considered the main driving force of GC variation [12,13,14]. gBGC occurs in recombination during meiosis, wherein the heteroduplex is formed between different parental alleles. In this condition, the mismatch repair favors GC over AT bases [15].

Until recently, what we knew about GC content variation and its potential mechanisms came primarily from genomic GC, leaving local GC variation largely unknown. It has long been known that variation in GC content among organisms could have a strong impact on the reconstruction of evolutionary phylogenies [16,17,18]. This is because the tree-building algorithm often groups together unrelated species with similar GC content [19]. This issue sparked our interest in exploring the GC variation of the internal transcribed spacer 2 (ITS2), a widely used phylogenetic marker for both DNA barcoding and plant systematics [20,21,22,23]. As a nuclear region, ITS2 tandemly repeats hundreds to thousands of copies at one or more chromosomal locations [20]. After concerted evolution via unequal crossing over or gene conversion during the repeating recombination event [24], these copies become homogenized within a species. Given that both ITS2’s concerted evolution and the gBGC occur in the same recombination event, the more homogeneous the ITS2 sequences become, the more GC content they comprise.

*Corydalis*, comprising over 500 species, is a typical evolutionary complex group that exhibits an extensive range of morphology and habitats [25]. As many of the *Corydalis* species have been used as traditional medicines in East Asia, ITS2, an official DNA barcode for medicinal plants, has been increasingly studied in *Corydalis* [26,27,28]. It was found that the concerted evolution of *Corydalis* ITS2 is incomplete, and different numbers of heterozygous ITS2 copies were observed among *Corydalis* samples [26,27]. This multi-copy nature of *Corydalis* ITS2 makes it possible to test the gBGC hypothesis based on the correlation between ITS2 heterozygosity and GC content. In particular, our previous study demonstrated that *Corydalis* ITS2 evolves in the context of secondary structure, i.e., substitutions on one side of the double-stranded regions (stems) are compensated by substitutions on the other side; thus, this study extended our understanding of ITS2’s evolution from a sequence to structure view [27]. Given that GC content contributes greatly to the stability of the secondary structure, it is of interest to explore whether GC evolves toward GC content enrichment. If so, is it affected by the gBGC mechanism?

In this study, high-throughput sequencing provided enough sequences of ITS2 from 29 closely related species to test this correlation between ITS2 heterozygosity and GC content and thus verify the gBGC hypothesis. Furthermore, after transcription, ITS2 has a recognized secondary structure [24], which is highly conserved throughout the eukaryote [29,30,31]. Thus, the availability of ITS2 structural information could facilitate exploration of the correlation between GC content and thermal stability. We assessed the relationship between GC content and thermodynamic stability by virtue of ITS2’s recognized secondary structure. As ITS2 is a widely used phylogenetic marker, our findings on ITS2 GC content variation could greatly improve its evolutionary modelling and facilitate its phylogenetic use.

## 2. Results

### 2.1. GC Content Differs Significantly between ITS2 and 5.8S Region

**Figure 1:**
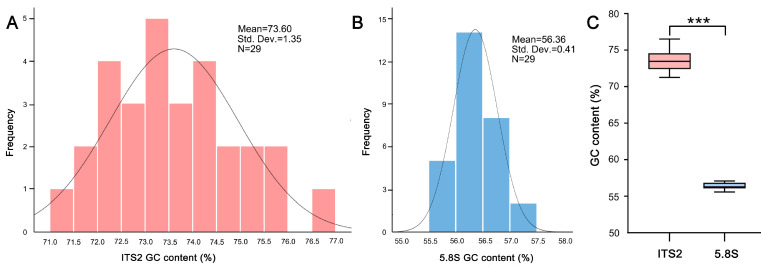
Comparison of GC content between ITS2 and 5.8S among *Corydalis* lineages. (**A**,**B**) Histogram of ITS2 and 5.8S GC contents across 29 sampled species showing their distribution ranges. (**C**) Box plots showing the different GC content values between ITS2 and 5.8S. *** *p* < 0.001.

After ambiguous reads were denoised and the reads with a single sequence were removed, 4 to 190 ITS2 variants and 34 to 578 5.8S variants were identified among the genomes of our 29 plants (Table S1). The GC content of ITS2 ranged from 71.26% (*C. oxypetala*) to 76.50% (*C. decumbens*), with a mean value of 73.60%. The GC distribution of the 29 species was plotted as a histogram, and a Shapiro–Wilk test revealed that it conforms to a normal distribution (*p* > 0.05; Figure 1A). By contrast, that of 5.8S ranged from 55.58% (*C. fangshanensis*) to 57.08% (*C. laucheana*), with a mean value of 56.36%. A Shapiro–Wilk test of the 5.8S GC distribution in a histogram also showed a normal distribution (*p* > 0.05; Figure 1B). Notably, despite adjacent regions, we found the ITS2 GC content was always higher than that of the 5.8S for each of the 29 species, averaging 131% of that of the 5.8S (Table S2). Furthermore, the GC content variation of ITS2 (GC-rich) was larger than that of 5.8S (GC-poor; Figure 1). Taken together, ITS2 has undergone GC-biased evolution (Figure 1C).

### 2.2. Comparison of GC and GC* Content between ITS2 Paired and Unpaired Regions

**Figure 2:**
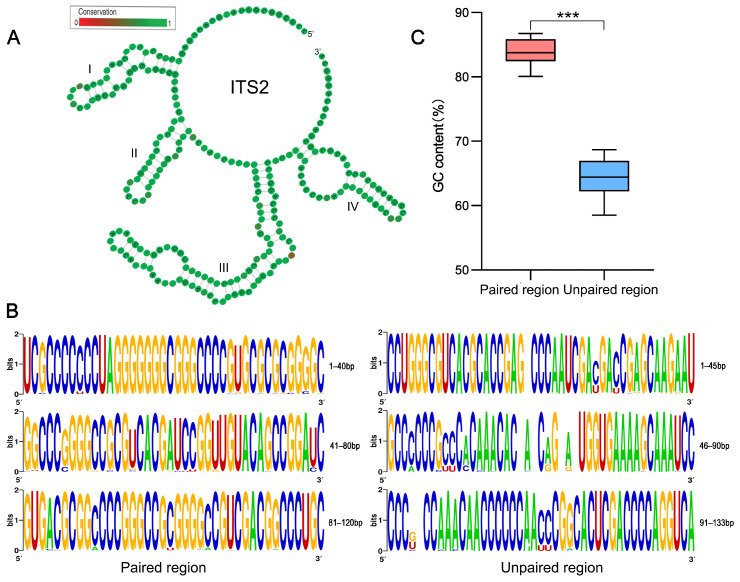
GC distribution in ITS2 secondary structure. (**A**) An example of ITS2 consensus secondary structure from *C. rheinbabeniana*. The four stems are labelled I–IV. The degree of conservation over the entire sequences is displayed in color grades from green (conservative) to red (variable). (**B**) ITS2 sequence logo of *C. rheinbabeniana* for visualization of base composition in different ITS2 sequence–structure partitions. The overall height of letter stack in each position indicates the sequence conservation (measured in bits), the height of letter within the stack represents the relative frequency of the bases at that position. (**C**) The statistics of GC contents in ITS2 sequence–structure partitions among 29 investigated species. *** *p* < 0.001.

The ITS2 secondary structure predicted in each species showed a typical ‘four-fingered hand’ form. Their consensus secondary structure had four stems, of which stem III was always the longest. The stem II contained a pyrimidine–pyrimidine bulge, and the loop between stems had a pronounced adenine bias (Figure 2A). We found that the length of *Corydalis* ITS2 ranged from 240 to 277 bp, with an average of 119 bp in paired regions and 139 bp in unpaired regions. The GC content in the paired region (GCp) was always higher than that in the unpaired region (GCup) for each ITS2 sequence–structure matrix in our study (Figure 2B). Taken across all 29 ITS2 sequence–structure matrices, the average GCp was 130% that of the average GCup (83.79% vs. 64.43%; Table S3, Figure 2C). Furthermore, for 90% of the ITS2 sequence–structure matrix, the homogeneity of GC content in the paired region is higher than that of the unpaired region (average SD: 0.65% vs. 1.06%; Table S3).

**Figure 3:**
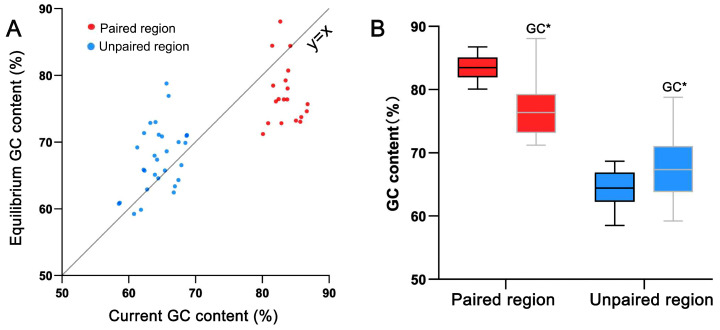
Comparison of current and the equilibrium GC contents between the paired and unpaired regions of ITS2 secondary structures. (**A**) A scatter plot of current–equilibrium GC content showing the distinct evolutionary trend of GC between the between the paired and unpaired ITS2 regions among 19 investigated samples. (**B**) The statistics of current and the equilibrium GC contents between the paired and unpaired ITS2 regions.

MCMC analysis showed that a total of 19 matrixes with paired regions can be inferred to be in the equilibrium state (Table S4). The majority of the GC* in the unpaired regions was found to be higher than the current GC (67.26% vs. 63.79%; Table S4), indicating that the substitution pattern tends to increase GC content. In contrast, for the paired regions, the majority of the GC* was lower than the current GC (77.16% vs. 83.48%; Table S4), indicating that the substitution pattern tends to reduce GC content. Obviously, the paired and unpaired regions have opposing GC evolutionary trends (Figure 3).

### 2.3. Correlation between GC Content and Sequence Homogeneity

**Figure 4:**
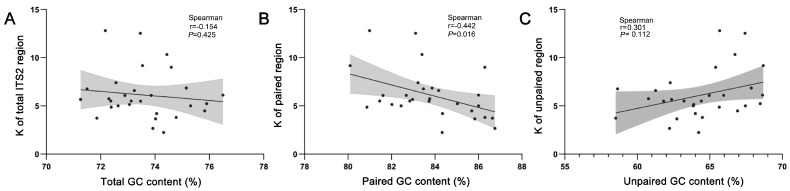
Correlations between the average number of nucleotide differences (K) and GC content among the 29 ITS2 sequence–structure matrices. Each point corresponds to the average GC content of the ITS2 alleles. The line of regression was calculated by the Spearman’s correlation, the error bands represent 95% confidence intervals based on a binomial model. (**A**) Comparison of the GC content and the K value of the whole ITS2 sequence. (**B**,**C**) Comparison of the GC content and the K value of the ITS2 paired and unpaired regions.

We assumed that sequence homogenization might be accompanied by a GC increase in recombination events. Indeed, we found that ITS2 sequences with a low average number of nucleotide differences (K) were always GC-rich. For example, the GC was 74.32% in the K-lowest *C. fangshanensis* (K = 2.24), compared to 72.17% in the K-highest *C. kokiana* (K = 12.80). Taken across all 29 species alignments, we calculated the K value and the ITS2 GC content in each species and found GC generally increases as K decreases (Figure 4A). This result supports the gBGC hypothesis; however, some other factors could also affect GC content, since the negative correction was very weak (r = −0.154, *p* = 0.425; Figure 4A). Notably, we found that gBGC in the paired region was very evident (Spearman, r = −0.442, *p* = 0.016; Figure 4B), while the unpaired region showed an opposing trend (Spearman, r = 0.301, *p* = 0.112; Figure 4C). Taken together, it is probably not merely gBGC involved in ITS2 GC enrichment.

### 2.4. Base Pair Transformations in ITS2 Paired Regions

**Figure 5:**
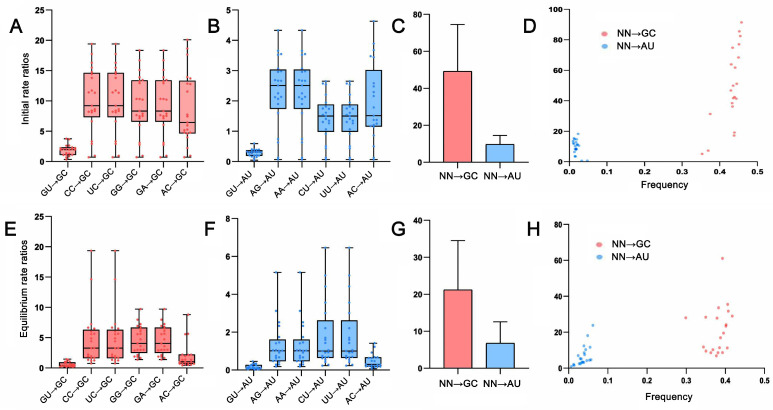
Comparison of the base pair transformations to GC and AU among 29 ITS2 transition–rate matrices under the best-fit RNA substitution models. For each matrix, the transition rate was normalized so that its average substitution rate is 1.0. (**A**–**D**) Base pair transformations derived from an initial state; (**A**,**B**) The relative rates of the six possible transformations to GC and AU, respectively. (**C**) Comparison of the total formation rates to GC and AU base pairs. (**D**) Frequency–mutability scatter plot of formation rates to GC and AU base pairs showing an increased GC fixation with GC enrichment. (**E**–**H**) Base pair transformations derived from the equilibrium state. (**E**,**F**) The relative rates of the six possible transformations to GC and AU, respectively. (**G**) Comparison of the total formation rates to GC and AU base pairs. (**H**) Frequency–mutability scatter plot of formation rates to GC and AU base pairs showing an increased GC fixation with GC enrichment.

Given the basic assumption that the substitution process is constant within a given lineage, we can use an evolutionary model to infer the substitution process, including base frequency and rate parameters. We found that the most common best-fit RNA substitution model was RNA16D (55%), followed by RNA16E (10%), RNA7G (10%), and RNA7E (10%), none of which allow for double substitutions of both nucleotides in a base pair. In other words, base pair substitution occurred mainly through an intermediate state, i.e., GC-GU-AU. In total, there were six one-site substitutions from intermediates to GC and AU, respectively (Figure 5). The rate matrix of an initial state showed that the substitution rates from the intermediate base pairs to GC were more or less equal, except for the relatively low rate of GU→GC and GU→AU (Figure 5A,B). However, the substitution rate from intermediate to GC was always higher than that to AU (Table S5). Taken together, the GC generation rate was 506% higher than that of AU (Figure 5C). Notably, we found that the higher GC generating rate was always accompanied by the higher GC frequency, indicating that the fast GC base pair substitution enhanced the probability of GC allele fixation compared to AU (Figure 5D). When the substitution was inferred to be at equilibrium, the base pair substitution rates decreased, but the evolutionary trends remained unchanged; the GC generating rate remained 378% higher than that of AU, and the probability of GC allele fixation remained increased (Figure 5E–H, Table S6).

**Figure 6:**
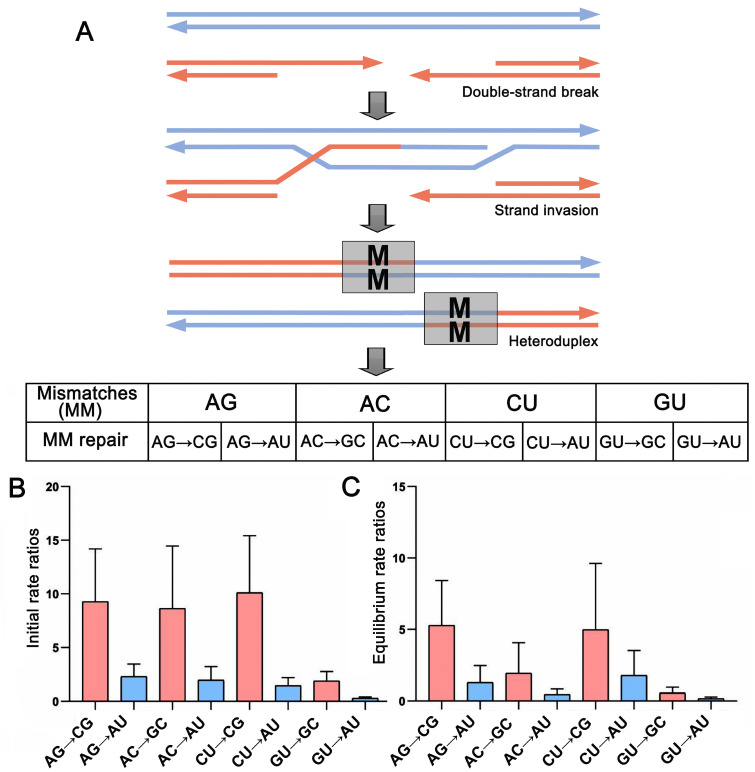
Simulation of mismatch base pair transformation in recombination. (**A**) A schematic representation of gene conversion meiotic recombination. The double-strand always breaks during meiosis. Then, a heteroduplex forms after the single-stranded DNA invades the homologous sequence, wherein the possible four mismatches can be repaired by changing one side of the nucleotides. (**B**,**C**) Comparison of mismatch base pair transformations showing a GC-biased gene conversion in both (**B**) initial and (**C**) equilibrium states. The base pair transformation rate derived from 29 ITS2 transition–rate matrices under the best-fit RNA substitution models.

There were a total of four possible mismatched base pairs (MM:AG\AC\CU\GU) in the heterozygous sites during recombination, all of which can change into the stable GC or the counterpart AU, e.g., AG→CG, AG→AU (Figure 6A). In sum, there were a total of eight (four pairs) base pair changes in the heterozygous sites. The rate matrix of an initial state showed that the substitution rate from the MM to GC was always higher than that of the AU for all four pairwise MM transformations, averaging 480% of the AU (Figure 6B, Table S7). When the substitution was inferred to be at equilibrium, the MM substitution rates decreased, but the substitution rate from MM to GC was still higher than that of AU, averaging 375% of that AU (Figure 6C, Table S8). Clearly, there was an MM conversion bias toward the GC base pair during the mismatch repair.

## 3. Discussion

The spatial heterogeneity of genomic base composition, initially known as isochores in mammalian genomic landscapes, has been increasingly understood by the gBGC model, based on the fact that regions subject to fast recombination are always GC-rich [13]. However, the recombination occurs dispersedly in hotspots which account for merely 3% of the human genome [32]. In addition, recombination hotspots were not conserved, even between closely related organisms [33,34], indicating the relatively short lifespans of recombination hotspots. This spatiotemporal heterogeneity of recombination always causes confusion when assessing correlations between recombination and GC-contents in the large-scale genomic region [10,12]. In this study, we alternatively selected ITS2, a very short rDNA region that evolves under frequent recombination among all organisms [20]. To take into account all GC-biased polymorphisms, we extracted all possible ITS2 copies from 29 closely related species via high-throughput sequencing. As a result, this study represents a test of the gBGC hypothesis at short genomic and time scales.

Our results showed that both the GC contents and their variation ranges in the ITS2 region are significantly higher than those of 5.8S (Figure 1). Obviously, these striking differences demonstrate that ITS2 has undergone GC-biased evolution. Given that 5.8S is a structural gene of the ribosome and thus is subject to strong functional constraints against substitution, in contrast, the ITS2 region is not directly involved in the ribosome structure; therefore, it is less constrained by selection [24], making it more likely to accumulate GC polymorphisms.

ITS2 is a well-known nrDNA region that undergoes concerted evolution due to the high rates of local recombination [20]. Consequently, the distinct intragenic ITS2 copies gradually become homogeneous [35]. Under the gBGC model, the genomic regions with high local rates of recombination also evolve toward high GC content [12,36,37]. Taken together, we hypothesized that the homogeneous ITS2 could be GC-rich. In accordance with this prediction, we found that the ITS2 GC content increased as the ITS2 sequence polymorphism decreased, indicating the occurrence of gBGC (Figure 4A). In addition, direct evidence for gBGC has been observed in the base pair transformations to GC from AU in the paired regions (Figure 5). Furthermore, transformations in mismatches to GC/CG are clearly higher than those in AU/UA (Figure 6). Notably, these elevated GC transformations can strongly promote the probability of GC allele fixation. Again, these observations fit well with the gBGC hypothesis that transformations favor GC over the AT base pair when mismatch repair occurs in the recombination heteroduplex [15,36]. Given that recombination rates vary greatly among closely related organisms [33,34], the gBGC model may explain why the GC content is heterogeneous on the *Corydalis* phylogeny (Figure S1).

A highlight of this study is the finding that the GC content of paired regions is significantly higher than that of unpaired regions, indicating that gBGC has been enhanced in ITS2 paired regions (Figure 2). Notably, GC* content shows an opposing trend of GC evolution between paired and unpaired regions: GC evolves toward GC increasing in unpaired regions, whereas GC decreases in paired regions (Figure 3). This result reveals that the present substitution pattern is very different from what it will be in the future. In other words, other driving forces in addition to gBGC have maintained the current elevated GC content of paired regions.

Previous studies show that ITS2 rDNA folds and functions in vivo in the form of secondary structure [38]. Despite a rapid rate of nucleotide substitutions, all our investigated ITS2 sequences show a conserved ‘four-fingered hand’ secondary structure and some core motifs that are shared within angiosperms [29,30,31], indicating that selective constraint has acted on the ITS2 secondary structure. Given that the secondary structure is maintained through base pair interactions, GC base pairs have higher thermodynamic stability than those of AU pairs. It is reasonable to hypothesize that GC content has been selected for the thermodynamic stability of the ITS2 secondary structure [39]. Taken together, we assumed that the gBGC would elevate GC content and the structural stability would enhance GC selection. This assumption was supported by comparing the unpaired regions, wherein GC elevation is almost unrelated to gBGC in the absence of structural constraint (Figure 4C).

Since the rDNA ITS2/ITS are the most widely used phylogenetic markers for phylogenetic inference [21,22,23], their GC compositional heterogeneity should be taken seriously. Both theoretical and empirical studies have increasingly shown that changes in nucleotide frequency among taxa could mislead phylogenetic inference because unrelated lineages with similar GC composition are often found to cluster together, irrespective of their true evolutionary relationships [16,17,18,19]. We observed that GC contents and their evolutionary trends are strikingly different between the paired and unpaired regions within the ITS2 secondary structure (Figure 2 and Figure 3), highlighting that using a single model to account for the whole ITS2 evolution may fail to accurately portray locus-specific evolutionary patterns. Several evolutionary models have been proposed to account for the compositional heterogeneity in phylogenetic inference, among which partitioning is by far the most effectively used approach [40,41,42]. Conventionally, constructing partitions requires some biological knowledge about the sequences as an a priori definition of appropriate groups of sites [43,44]. The strong nucleotide composition bias in our results indicates that the paired and unpaired nucleotide states can be an a priori definition of ITS2 partitioning. Consistent with this idea, we found that for all 29 *Corydalis* matrices, the mixed models (DNA models for unpaired regions and RNA-base pair models for paired regions) all outperformed the DNA-only models, according to the Akaike information criterion (AIC) [45].

Although the application of partitioning is theoretically justified, more partitions with more complex models mean more parameters are estimated, causing the concern of over-parameterization [46]. Recent empirical studies have found that using single DNA models for unpartitioned sequences violated ITS2 evolution but was insufficient to mislead phylogenetic inference within closely related lineages, wherein few base pair substitutions are observed in paired regions [27,47]. Taken together, a threshold for the magnitude of model violations should be considered before using the partition method for ITS2 phylogenetic inference.

## 4. Materials and Methods

### 4.1. Taxon Sampling and Sequence Acquisition

A total of 29 species of *Corydalis* were used in this study, including 28 field-collected individuals for this and our phylogenomic study and one species with genomic data in GenBank (Table S1). These species represent 19 closely related sections as delimited by the Flora of China and recent Corydalis phylogeny [48]. The total genomic DNA of each species was extracted by the modified CTAB method [49], and the quality control was carried out by 1% agarose gel electrophoresis and Qubit 3.0. Then, DNA libraries were constructed according to the DNA short-insert library construction protocol and sequenced on the DNBseq platform in PE150 mode (insert size = 300 bp). The amount of sequencing data for each species was about 6–8 Gb, covering about 40× of the nuclear genome.

The raw data were surveyed using FastQC v.0.11.8 (https://www.bioinformatics.babraham.ac.uk/projects/fastqc/ (accessed on 20 October 2021)) and then filtered to remove the low-quality bases and adapter sequences using Trimmomatic v.0.39 [50]. Due to the genetic difference of nuclear ribosomal DNA (nrDNA), which is large between and small within species’ alleles, we first employed GetOrganelle v.1.7.5 to assemble the main type of nrDNA in each species [51], and then they were treated as reference sequences to extract nrDNA reads as much as possible using bowtie2, with the sequencing depth being calculated by samtools v.1.9 [52,53]. The extracted nrDNA reads were assembled using FLASH v.1.2.11; the length of short reads was extended by overlapping and merging paired-end reads [54]. Finally, BLAST v.2.7.1 was applied to obtain all ITS2/5.8S alleles from the assembled sequences, and then MAFFT v.7.407 and SeqKit v.2.2.0 were used to calculate the number of each allele [55,56,57]. The allele associated with a single sequence was removed from downstream analyses to eliminate the possible artificial allele types generated from sequencing and assembly.

### 4.2. ITS2 Secondary Structure Prediction and Partition

The individual ITS2 secondary structure was predicted through the online service of the ITS2 database (http://its2.bioapps.biozentrum.uni-wuerzburg.de/ (accessed on 10 February 2022)), wherein plenty of existing ITS2 sequences with their modeled structures were used for homology prediction [58]. Then, all ITS2 sequences with their structures (Vienna format) were integrated into a sequence–structure matrix and aligned using 4SALE1.7 [59]. 4SALE is designed to synchronously handle sequence and secondary structure. It provides an editable consensus secondary structure and a new method of simultaneous visualization and editing of sequences. We adjusted the consistency of the consensus secondary structure to 0.70, then converted the graphic information into Vienna format. Based on the consensus secondary structure, we partitioned the ITS2 matrix into paired and unpaired regions and performed phylogenetic analyses both separately and in combination.

### 4.3. Inferring Substitution Parameters of ITS2 Sequence Structure

In ITS2 phylogenetic analyses, DNA/RNA mixed substitution models were used to account for the differences in substitution patterns between paired and unpaired ITS2 regions. A model Perl script (model_selection.pl) from PHASE package 3.0 was performed to infer ITS2 substitution [60]. This Perl script includes two DNA models (HKY85 and REV) for unpaired regions and 16 types of RNA models (e.g., RNA16D, RNA16E, RNA7G and RNA7E) for paired regions [60,61]. A likelihood correction method was used to address the different numbers of parameters between the four-, seven-, and 16-state models, and thus to facilitate best model selection among different model types according to the AICc values [60]. Bayesian MCMC phylogenetic analyses were performed using the mcmcPHASE program from the PHASE package based on the best-fit mixed models and three other files: the ITS2 sequence alignment file, the ITS2 consensus secondary structure file, and an input NJ tree file. The MCMC analysis was run for 1,000,000 generations, sampling every 100 generations, with a burn-in of 3000 (30%) trees. The iteration of each run was increased by 1,000,000, 4,000,000, and 7,000,000 iterations, respectively, until equilibrium reached the convergence state (in which the iteration increase does not change the value of the substitution model parameters). Then, rate matrices of both the initial tree and the best-fit tree were inferred by the mcmcsummarize program in the PHASE package, from which the relative base pair transformation rates at equilibrium were obtained.

The equilibrium GC (GC*) content was used to assess the GC evolutionary trend, based on the assumption that GC* can be obtained when sequences evolve convergently at the stationary (equilibrium) state under the constant patterns of substitution. In this state, the GC* value can be calculated as the percentage of the AT→GC substitution rate among all AT→GC and GC→AT substitution rates, i.e., GC* = r_AT→GC_/(r_AT→GC_) + (r_AT→GC_) [62]. We used the maximum likelihood approach and the program mcmcsummarize in the PHASE package to calculate GC* values in a given species matrix.

### 4.4. Calculation of GC Content and Sequences Homogenization

For each species alignment, the 5.8S and ITS2 sequences and their paired and unpaired regions were separately analyzed by MEGA11 to calculate their GC content [63]. The same data were also analyzed by DnaSP6 to assess the sequence homogenization by calculating the average value of nucleotide difference among each species (K value) [64].

### 4.5. Phylogenetic and Statistic Analyses

We performed BI inference using MrBayes version 3.2 [65]. Two independent runs that each consisted of four MCMC chains were run for 1,000,000 generations each, sampling every 100 generations. The initial 3000 sampled trees were discarded as burn-in, and the remaining trees were used to construct the 50% majority rule consensus tree. The tree was edited and GC annotated using the Interactive Tree Of Life (iTOL: https://itol.embl.de/ (accessed on 20 March 2022)). The data, including GC and GC* contents, base pair transformation rates, and K values, were statistically analyzed by Excel, SPSS 26.0, and GraphPad Prism 8 software. In Excel, the data were extracted and sorted out after the original matrix was classified and summarized. In SPSS, the Shapiro–Wilk method and a histogram fitting a normal curve were used to test the normality of the data, and a Spearman correlation analysis was used to perform a correlation test. In GraphPad Prism, a histogram, box diagram, and scatter plot were used to analyze the difference between the comparable datasets. The relative frequency of bases at every position of the consensus secondary structure was graphically presented by the sequence logo, which was produced on the WebLogo website [66].

## Data Availability

The ITS2 sequence data and the sequence-structure alignment of each sample are available at: https://figshare.com/articles/dataset/Structure-based_GC_investigation_shed_new_light_on_ITS2_evolution_in_Corydalis_species/21980624 (accessed on 1 April 2023).

## Supplementary Materials

The supporting information can be downloaded at: https://www.mdpi.com/article/10.3390/ijms24097716/s1.

## References

[1] Li X.Q., Du D.L. (2014). Variation, evolution, and correlation analysis of C plus G content and genome or chromosome size in different kingdoms and phyla. PLoS ONE.

[2] Hershberg R., Petrov D.A. (2010). Evidence That Mutation Is Universally Biased towards AT in Bacteria. PLoS Genet..

[3] McCutcheon J.P., Moran N.A. (2012). Extreme genome reduction in symbiotic bacteria. Nat. Rev. Microbiol..

[4] Smarda P., Knapek O., Brezinova A., Horova L., Grulich V., Danihelka J., Vesely P., Smerda J., Rotreklova O., Bures P. (2019). Ge-nome sizes and genomic guanine plus cytosine (GC) contents of the Czech vascular flora with new estimates for 1700 species. Preslia.

[5] Serres-Giardi L., Belkhir K., David J., Glémin S. (2012). Patterns and Evolution of Nucleotide Landscapes in Seed Plants. Plant Cell.

[6] Singh R., Ming R., Yu Q. (2016). Comparative Analysis of GC Content Variations in Plant Genomes. Trop. Plant Biol..

[7] Eyre-Walker A., Hurst L.D. (2001). The evolution of isochores. Nat. Rev. Genet..

[8] Parvathy S.T., Udayasuriyan V., Bhadana V. (2022). Codon usage bias. Mol. Biol. Rep..

[9] Plotkin J.B., Kudla G. (2011). Synonymous but not the same: The causes and consequences of codon bias. Nat. Rev. Genet..

[10] Muyle A., Serres-Giardi L., Ressayre A., Escobar J., Glémin S. (2011). GC-Biased Gene Conversion and Selection Affect GC Content in the Oryza Genus (rice). Mol. Biol. Evol..

[11] Wolfe K.H., Sharp P.M., Li W.-H. (1989). Mutation rates differ among regions of the mammalian genome. Nature.

[12] Duret L., Galtier N. (2009). Biased Gene Conversion and the Evolution of Mammalian Genomic Landscapes. Annu. Rev. Genom. Hum. Genet..

[13] Eyre-Walker A. (1993). Recombination and mammalian genome evolution. Proc. R. Soc. Lond. Ser. B Biol. Sci..

[14] Lassalle F., Périan S., Bataillon T., Nesme X., Duret L., Daubin V. (2015). GC-Content Evolution in Bacterial Genomes: The Biased Gene Conversion Hypothesis Expands. PLoS Genet..

[15] Marais G. (2003). Biased gene conversion: Implications for genome and sex evolution. Trends Genet..

[16] Foster P.G. (2004). Modeling Compositional Heterogeneity. Syst. Biol..

[17] Gruber K.F., Voss R.S., Jansa S.A. (2007). Base-compositional heterogeneity in the RAG1 locus among didelphid marsupials: Implications for phylogenetic inference and the evolution of GC content. Syst. Biol..

[18] Liu Y.Q., Song F., Jiang P., Wilson J.J., Cai W.Z., Li H. (2018). Compositional heterogeneity in true bug mitochondrial phylogenomics. Mol. Phylogenetics Evol..

[19] Mooers A.Ø., Holmes E.C. (2000). The evolution of base composition and phylogenetic inference. Trends Ecol. Evol..

[20] Álvarez I., Wendel J.F. (2003). Ribosomal ITS sequences and plant phylogenetic inference. Mol. Phylogenetics Evol..

[21] Chen S.L., Yao H., Han J.P., Liu C., Song J.Y., Shi L.C., Zhu Y.J., Ma X.Y., Gao T., Pang X.H. (2010). Validation of the ITS2 Region as a Novel DNA Barcode for Identifying Medicinal Plant Species. PLoS ONE.

[22] Li D.-Z., Gao L.-M., Li H.-T., Wang H., Ge X.-J., Liu J.-Q., Chen Z.-D., Zhou S.-L., Chen S.-L., Yang J.-B. (2011). Comparative analysis of a large dataset indicates that internal transcribed spacer (ITS) should be incorporated into the core barcode for seed plants. Proc. Natl. Acad. Sci. USA.

[23] Qin Y., Li M., Cao Y., Gao Y., Zhang W. (2017). Molecular thresholds of ITS2 and their implications for molecular evolution and species identification in seed plants. Sci. Rep..

[24] Zhang W., Tian W., Gao Z.P., Wang G.L., Zhao H. (2020). Phylogenetic Utility of rRNA ITS2 Sequence-Structure under Functional Constraint. Int. J. Mol. Sci..

[25] Xu X.D., Wang D. (2021). Comparative Chloroplast Genomics of Corydalis Species (Papaveraceae): Evolutionary Perspectives on Their Unusual Large Scale Rearrangements. Front. Plant Sci..

[26] Jiang L., Li M.H., Zhao F.x., Chu S.S., Zha L.P., Xu T., Peng H.S., Zhang W. (2018). Molecular Identification and Taxonomic Implication of Herbal Species in Genus Corydalis (Papaveraceae). Molecules.

[27] Li M.H., Zhao H., Zhao F.X., Jiang L., Peng H.S., Zhang W., Simmons M.P. (2019). Alternative analyses of compensatory base changes in an ITS2 phylogeny of Corydalis (Papaveraceae). Ann. Bot..

[28] Ren F.-M., Wang Y.-W., Xu Z.-C., Li Y., Xin T.-Y., Zhou J.-G., Qi Y.-D., Wei X.-P., Yao H., Song J.-Y. (2019). DNA barcoding of *Corydalis*, the most taxonomically complicated genus of Papaveraceae. Ecol. Evol..

[29] Coleman A.W. (2003). ITS2 is a double-edged tool for eukaryote evolutionary comparisons. Trends Genet..

[30] Hershkovitz M.A., Zimmer E.A. (1996). Conservation patterns in angiosperm rDNA ITS2 sequences. Nucleic Acids Res..

[31] Schultz J., Maisel S., Gerlach D., Müller T., Wolf M. (2005). A common core of secondary structure of the internal transcribed spacer 2 (ITS2) throughout the Eukaryota. RNA.

[32] Myers S., Bottolo L., Freeman C., McVean G., Donnelly P. (2005). A Fine-Scale Map of Recombination Rates and Hotspots Across the Human Genome. Science.

[33] Coop G., Wen X., Ober C., Pritchard J.K., Przeworski M. (2008). High-Resolution Mapping of Crossovers Reveals Extensive Variation in Fine-Scale Recombination Patterns Among Humans. Science.

[34] Stapley J., Feulner P.G.D., Johnston S.E., Santure A.W., Smadja C.M. (2017). Variation in recombination frequency and distribution across eukaryotes: Patterns and processes. Philos. Trans. R. Soc. B: Biol. Sci..

[35] Naidoo K., Steenkamp E.T., Coetzee M.P.A., Wingfield M.J., Wingfield B.D. (2013). Concerted Evolution in the Ribosomal RNA Cistron. PLoS ONE.

[36] Galtier N. (2003). Gene conversion drives GC content evolution in mammalian histones. Trends Genet..

[37] Mugal C.F., Weber C.C., Ellegren H. (2015). GC-biased gene conversion links the recombination landscape and demography to genomic base composition GC-biased gene conversion drives genomic base composition across a wide range of species. BioEssays.

[38] Fromm L., Falk S., Flemming D., Schuller J.M., Thoms M., Conti E., Hurt E. (2017). Reconstitution of the complete pathway of ITS2 processing at the pre-ribosome. Nat. Commun..

[39] Higgs P.G. (2000). RNA secondary structure: Physical and computational aspects. Q. Rev. Biophys..

[40] Blair C., Murphy R.W. (2011). Recent trends in molecular phylogenetic analysis: Where to next?. J. Hered..

[41] Kainer D., Lanfear R. (2015). The Effects of Partitioning on Phylogenetic Inference. Mol. Biol. Evol..

[42] Lanfear R., Frandsen P.B., Wright A.M., Senfeld T., Calcott B. (2017). PartitionFinder 2: New Methods for Selecting Partitioned Models of Evolution for Molecular and Morphological Phylogenetic Analyses. Mol. Biol. Evol..

[43] Crotty S.M., Holland B.R. (2022). Comparing partitioned models to mixture models: Do information criteria apply?. Syst. Biol..

[44] Rota J., Malm T., Chazot N., Peña C., Wahlberg N. (2018). A simple method for data partitioning based on relative evolutionary rates. PeerJ.

[45] Akaike H. (1974). A new look at the statistical model identification. IEEE Trans. Autom. Control.

[46] Fan Y., Wu R., Chen M.-H., Kuo L., Lewis P.O. (2011). Choosing among Partition Models in Bayesian Phylogenetics. Mol. Biol. Evol..

[47] Cao R.X., Tong S.Y., Luan T.J., Zheng H.Y., Zhang W. (2022). Compensatory base changes and varying phylogenetic effects on an-giosperm ITS2 genetic distances. Plants.

[48] Xu X.D., Li X.X., Wang D. (2022). New Insights into the Backbone Phylogeny and Character Evolution of Corydalis (Papaveraceae) Based on Plastome Data. Front. Plant Sci..

[49] Porebski S., Bailey L.G., Baum B.R. (1997). Modification of a CTAB DNA extraction protocol for plants containing high polysaccha-ride and polyphenol components. Plant Mol. Biol. Report..

[50] Bolger A.M., Lohse M., Usadel B. (2014). Trimmomatic: A flexible trimmer for Illumina sequence data. Bioinformatics.

[51] Jin J.-J., Yu W.-B., Yang J.-B., Song Y., Depamphilis C.W., Yi T.-S., Li D.-Z. (2020). GetOrganelle: A fast and versatile toolkit for accurate de novo assembly of organelle genomes. Genome Biol..

[52] Langmead B., Salzberg S.L. (2012). Fast gapped-read alignment with Bowtie 2. Nat. Methods.

[53] Li H., Handsaker B., Wysoker A., Fennell T., Ruan J., Homer N., Marth G., Abecasis G., Durbin R., 1000 Genome Project Data Processing Subgroup (2009). The Sequence Alignment/Map format and SAMtools. Bioinformatics.

[54] Magoč T., Salzberg S.L. (2011). FLASH: Fast length adjustment of short reads to improve genome assemblies. Bioinformatics.

[55] Camacho C., Coulouris G., Avagyan V., Ma N., Papadopoulos J., Bealer K., Madden T.L. (2009). BLAST plus: Architecture and applications. BMC Bioinform..

[56] Nakamura T., Yamada K.D., Tomii K., Katoh K. (2018). Parallelization of MAFFT for large-scale multiple sequence alignments. Bioinformatics.

[57] Shen W., Le S., Li Y., Hu F.Q. (2016). SeqKit: A Cross-Platform and Ultrafast Toolkit for FASTA/Q File Manipulation. PLoS ONE.

[58] Selig C., Wolf M., Müller T., Dandekar T., Schultz J. (2008). The ITS2 Database II: Homology modelling RNA structure for molecular systematics. Nucleic Acids Res..

[59] Seibel P.N., Müller T., Dandekar T., Schultz J., Wolf M. (2006). 4SALE—A tool for synchronous RNA sequence and secondary structure alignment and editing. BMC Bioinform..

[60] Allen J.E., Whelan S. (2014). Assessing the State of Substitution Models Describing Noncoding RNA Evolution. Genome Biol. Evol..

[61] Savill N.J., Hoyle D.C., Higgs P.G. (2001). RNA sequence evolution with secondary structure constraints: Comparison of substitu-tion rate models using maximum-likelihood methods. Genetics.

[62] Sueoka N. (1962). On the genetic basis of variation and heterogeneity of DNA base composition. Proc. Natl. Acad. Sci. USA.

[63] Tamura K., Stecher G., Kumar S. (2021). MEGA11 molecular evolutionary genetics analysis version 11. Mol. Biol. Evol..

[64] Rozas J., Ferrer-Mata A., Sánchez-DelBarrio J.C., Guirao-Rico S., Librado P., Ramos-Onsins S.E., Sánchez-Gracia A. (2017). DnaSP 6: DNA Sequence Polymorphism Analysis of Large Data Sets. Mol. Biol. Evol..

[65] Ronquist F., Teslenko M., van der Mark P., Ayres D.L., Darling A., Höhna S., Larget B., Liu L., Suchard M.A., Huelsenbeck J.P. (2012). MrBayes 3.2: Efficient Bayesian Phylogenetic Inference and Model Choice across a Large Model Space. Syst. Biol..

[66] Crooks G.E., Hon G., Chandonia J.-M., Brenner S.E. (2004). WebLogo: A Sequence Logo Generator. Genome Res..

