# Supplementary material for: Structure-Based GC Investigation Sheds New Light on ITS2 Evolution in Corydalis Species
Source: Int J Mol Sci. 2023 Apr 23;24(9):7716. doi: 10.3390/ijms24097716 (PMC10178233; doi:10.3390/ijms24097716)
Supplement: Supplementary file 1 [file ijms-24-07716-s001.zip › ijms-2344800-supplementary.pdf]

Supplementary information

# Structure-based GC investigation shed new light on ITS2 evolution in *Corydalis* species

Qing Xian[1†], Suyin Wang[1†], Yanyan Liu[2], Shenglong Kan[3], Wei Zhang[1,*]

1 Marine College, Shandong University, Weihai, 264209, China
2 College of Plant Protection, Henan Agricultural University, Zhengzhou, 450002, China
3 Shenzhen Branch, Guangdong Laboratory of Lingnan Modern Agriculture, Genome Analysis Laboratory of the Ministry of Agriculture and Rural Affairs, Agricultural Genomics Institute at Shenzhen, Chinese Academy of Agricultural Sciences, Shenzhen, 518120, China
† Qing Xian and Suyin Wang contributed equally to this work.
***** Correspondence: Wei Zhang, wzhang@sdu.edu.cn

Tables S1. Samples and their ITS2/5.8S rDNA high-throughput sequencing information[a]

| Sample | Locality[b] | Species | Section | ITS2 copy # in HGS | | | 5.8S copy # in HGS | | |
|---|---|---|---|---|---|---|---|---|---|
| | | | | Total # | Singleton # | Depth | Total # | Singleton # | Depth |
| **WJL01** | Songlinkou, Balang MT., Xiaojin, Sichuan | *Corydalis flexuosa* | Sect. *Elatae* | 16 | 16 | 16976.90 | 1625 | 311 | 19430.40 |
| **WJL03** | Xueshanliang Viewing Platform, Chuanzhusi Town, Songpan, Sichuan | *Corydalis rheinbabeniana* | Sect. *Trachycarpae* | 135 | 115 | 21035.90 | 1063 | 281 | 18657.80 |
| **WJL04** | Menggu Village, Lixian, Sichuan | *Corydalis pingwuensis* | Sect. *Elatae* | 6 | 6 | 10334.40 | 1122 | 261 | 10838.80 |
| **WJL06** | Baima Snow MT., Deqin, Yunnan | *Corydalis lathyrophylla* | Sect. *Trachycarpae* | 135 | 109 | 19646.60 | 1704 | 460 | 19532.90 |
| **WJL07** | Baima Snow MT., Deqin, Yunnan | *Corydalis kokiana* | Sect. *Kokianae* | 5 | 5 | 11852.10 | 1459 | 378 | 14296.00 |
| **WJL08** | Baima Snow MT., Deqin, Yunnan | *Corydalis delphinioides* | Sect. *Davidianae* | 8 | 8 | 14993.40 | 1189 | 367 | 15165.60 |
| **WJL11** | Laoludu highway, Geza Township, Shangri-la,Yunnan | *Corydalis melanochlora* | Sect. *Clavatae* | 165 | 146 | 34346.90 | 3161 | 503 | 32452.40 |
| **WJL12** | Longyu Bay, Luanchuan, Henan | *Corydalis ophiocarpa* | Sect. *Cheilanthifoliae* | 30 | 26 | 13650.80 | 1308 | 317 | 12319.10 |
| **WJL13** | Top of Shika Snow MT., Shangri-La, Yunnan | *Corydalis pachypoda* | Sect. *Chrysocapnos* | 124 | 90 | 22557.70 | 1581 | 336 | 20248.70 |
| **WJL14** | Diaohaizi,Yajia ridge, Kangding, Sichuan | *Corydalis elata* | Sect. *Elatae* | 23 | 23 | 12846.20 | 1219 | 212 | 12120.90 |
| **WJL15** | Wanfoshan, Tongdao Dong Autonomous, Huaihua, Hunan | *Corydalis balansae* | Sect. *Sophorocapnos* | 88 | 64 | 34895.00 | 2842 | 578 | 30159.10 |
| **WJL16** | Daigu Temple,Diabe, Gansu | *Corydalis adunca* | Sect. *Strictae* | 4 | 4 | 39498.20 | 2721 | 571 | 33986.10 |
| **WJL17** | Longyu Bay, Luanchuan, Henan | *Corydalis caudata* | Sect. *Corydalis* | 89 | 74 | 31992.80 | 2479 | 480 | 32291.70 |
| **WJL18** | Ximatan, Cangshan, Dali, Yunnan | *Corydalis oxypetala* | Sect. *Fusiformes* | 4 | 4 | 4375.60 | 480 | 132 | 4511.60 |
| **WJL19** | Laoludu highway, Geza Township, Shangri-la,Yunnan | *Corydalis trachycarpa* | Sect. *Trachycarpae* | 148 | 116 | 15531.20 | 1186 | 263 | 14020.30 |

| | | | | | | | | | |
|---|---|---|---|---|---|---|---|---|---|
| **WJL20** | Laoludu highway, Geza Township, Shangri-la,Yunnan | *Corydalis eugeniae* | Sect. Trachycarpae | 180 | 136 | 23911.90 | 1715 | 338 | 22060.30 |
| **WJL22** | Xueshanliang Viewing Platform, Chuanzhusi Town, Songpan, Sichuan | *Corydalis curviflora* | Sect. *Curviflorae* | 12 | 12 | 16813.20 | 1824 | 345 | 19118.90 |
| **cyn17** | Lusi, Nanyang City, Henan | *Corydalis edulis* | Sect. *Aulacostigma* | 115 | 38 | 30007.20 | 3360 | 249 | 31175.40 |
| **cyn18** | Tongtianhe National Forest Park, Fengxian, Shaanxi | *Corydalis shensiana* | Sect. *Curviflorae* | 383 | 190 | 28945.70 | 2697 | 226 | 28669.30 |
| **CJL02** | Longbugou Zhonggu, Kangding, Sichuan | *Corydalis laucheana* | Sect. *Fumarioides* | 230 | 129 | 27493.70 | 2689 | 263 | 27573.90 |
| **CJL03** | Laoludu highway, Geza Township, Shangri-la,Yunnan | *Corydalis benecincta* | Set. *Benecinctae* | 39 | 21 | 3473.40 | 290 | 34 | 3325.40 |
| **CJL04** | Jinlan MT., National Forest Park, Xinxian, Xinyang, Henan | *Corydalis incisa* | Sect. Incisae | 187 | 96 | 25201.50 | 3976 | 236 | 26280.90 |
| **CJL05** | Alpine Botanical Garden, Lijiang, Yunnan | *Corydalis zhongdianensis* | Sect. *Fumarioides* | 58 | 31 | 6658.40 | 719 | 75 | 66290 |
| **CJL06** | Zhongshan Botanical Garden, Nanjing, Jiangsu | *Corydalis decumbens* | Sect. *Duplotuber* | 15 | 13 | 6819.70 | 598 | 78 | 7705.90 |
| **CJL07** | Bamei Town, Daofu, Sichuan | *Corydalis pseudosibirica* | Sect. *Fumarioides* | 114 | 34 | 19210.20 | 1316 | 118 | 178890 |
| **CJL08** | Beijing Botanical Garden,Beijing | *Corydalis bungeana* | Sect. *Chinenses* | 114 | 68 | 20865.90 | 1947 | 189 | 21049.80 |
| **CJL09** | Dongsong Town, Shangri-la, Ganzi, Sichuan | *Corydalis yui* | Sect. *Fumarioides* | 33 | 27 | 17242.50 | 678 | 87 | 17671.10 |
| **NJ01** | Zhongshan Botanical Garden, Nanjing, Jiangsu | *Corydalis sheareri* | Sect. *Asterostigmata* | 312 | 69 | 169633.00 | 9879 | 375 | 168492.10 |
| **Cfa** | NCBI | *Corydalis fangshanensis* | Sect. *Thalictrifoliae* | 38 | 14 | 6248.60 | 1140 | 106 | 6953.60 |

[a] The ITS2 sequence data and the sequence-structure alignment of each sample are posted at: https://figshare.com/articles/dataset/Structure-based_GC_investigation_shed_new_light_on_ITS2_evolution_in_Corydalis_species/21980624

[b] Fieldwork was all over China, the sampling localities included small site, county and province

Table S2. Comparison of GC content between ITS2 and 5.8S fragments

| **Species** | **ITS2** | | **5.8S** | |
|---|---|---|---|---|
| | GC content (%) | Standard Deviation | GC content (%) | Standard Deviation |
| *Corydalis yui* | 74.02 | 0.49 | 56.01 | 0.65 |
| *Corydalis flexuosa* | 73.54 | 1.02 | 56.14 | 0.86 |
| *Corydalis elata* | 75.15 | 0.77 | 56.81 | 0.82 |
| *Corydalis benecincta* | 73.06 | 0.42 | 55.86 | 0.53 |
| *Corydalis pseudosibirica* | 71.86 | 0.63 | 55.85 | 0.63 |
| *Corydalis melanochlora* | 73.86 | 0.67 | 56.84 | 0.87 |
| *Corydalis ophiocarpa* | 73.12 | 0.46 | 56.16 | 0.77 |
| *Corydalis trachycarpa* | 72.64 | 0.63 | 56.20 | 0.75 |
| *Corydalis pingwuensis* | 73.46 | 1.36 | 56.69 | 0.78 |
| *Corydalis adunca* | 75.31 | 0.35 | 56.30 | 0.84 |
| *Corydalis oxypetala* | 71.26 | 0.21 | 56.75 | 0.80 |
| *Corydalis sheareri* | 74.78 | 0.74 | 56.77 | 1.00 |
| *Corydalis curviflora* | 74.44 | 0.91 | 56.86 | 0.79 |
| *Corydalis laucheana* | 75.90 | 0.72 | 57.08 | 0.76 |
| *Corydalis incisa* | 73.47 | 0.58 | 56.81 | 0.65 |
| *Corydalis zhongdianensis* | 73.92 | 0.46 | 56.20 | 0.64 |
| *Corydalis bungeana* | 75.83 | 0.62 | 57.01 | 0.67 |
| *Corydalis shensiana* | 72.39 | 0.78 | 56.43 | 0.85 |
| *Corydalis rheinbabeniana* | 72.56 | 1.42 | 56.14 | 0.86 |
| *Corydalis pachypoda* | 72.37 | 0.64 | 56.22 | 0.77 |
| *Corydalis balansae* | 72.89 | 0.53 | 56.33 | 0.84 |
| *Corydalis lathyrophylla* | 72.30 | 0.73 | 56.24 | 0.80 |
| *Corydalis eugeniae* | 73.24 | 1.14 | 56.23 | 0.82 |
| *Corydalis caudata* | 71.50 | 0.79 | 56.28 | 0.79 |
| *Corydalis decumbens* | 76.50 | 0.92 | 55.70 | 0.69 |
| *Corydalis edulis* | 74.06 | 0.54 | 55.87 | 0.88 |
| *Corydalis fangshanensis* | 74.32 | 0.44 | 55.58 | 0.82 |
| *Corydalis delphinioides* | 74.60 | 0.62 | 56.31 | 0.78 |
| *Corydalis kokiana* | 72.17 | 1.02 | 56.81 | 0.82 |
| **Average** | 73.60 | 0.71 | 56.36 | 0.78 |

Table S3. Comparison of GC content between ITS2 paired and unpaired regions

| **Species** | **ITS2 paired regions** | | **ITS2 unpaired regions** | | **K value** |
|---|---|---|---|---|---|
| | GC content (%) | Standard Deviation | GC content (%) | Standard Deviation | |
| ***Corydalis yui*** | 85.83 | 0.49 | 62.73 | 0.96 | 3.66 |
| ***Corydalis flexuosa*** | 80.10 | 0.66 | 68.69 | 1.71 | 9.18 |
| ***Corydalis elata*** | 83.87 | 0.62 | 67.86 | 0.91 | 6.86 |
| ***Corydalis benecincta*** | 82.02 | 0.71 | 63.89 | 0.56 | 5.14 |
| ***Corydalis pseudosibirica*** | 86.64 | 0.47 | 58.51 | 1.02 | 3.73 |
| ***Corydalis melanochlora*** | 82.68 | 0.80 | 65.95 | 1.01 | 6.09 |
| ***Corydalis ophiocarpa*** | 82.84 | 0.42 | 64.41 | 0.86 | 5.51 |
| ***Corydalis trachycarpa*** | 82.44 | 0.74 | 63.86 | 0.92 | 5.01 |
| ***Corydalis pingwuensis*** | 83.11 | 0.45 | 67.43 | 1.99 | 12.53 |
| ***Corydalis adunca*** | 86.00 | 0.77 | 67.45 | 0.10 | 5.00 |
| ***Corydalis oxypetala*** | 82.97 | 0.37 | 62.36 | 0.21 | 5.67 |
| ***Corydalis sheareri*** | 86.30 | 0.54 | 64.49 | 1.16 | 3.81 |
| ***Corydalis curviflora*** | 83.40 | 0.97 | 66.74 | 1.75 | 10.33 |
| ***Corydalis laucheana*** | 85.03 | 0.56 | 68.48 | 1.17 | 5.23 |
| ***Corydalis incisa*** | 83.73 | 0.60 | 61.81 | 0.85 | 5.49 |
| ***Corydalis zhongdianensis*** | 86.76 | 0.51 | 62.22 | 0.68 | 2.67 |
| ***Corydalis bungeana*** | 85.69 | 0.57 | 66.91 | 1.01 | 4.48 |
| ***Corydalis shensiana*** | 80.87 | 0.74 | 65.65 | 1.21 | 4.87 |
| ***Corydalis rheinbabeniana*** | 83.22 | 0.99 | 62.27 | 1.98 | 7.40 |
| ***Corydalis pachypoda*** | 81.46 | 0.79 | 63.22 | 0.94 | 5.50 |
| ***Corydalis balansae*** | 81.61 | 0.49 | 64.96 | 0.84 | 6.08 |
| ***Corydalis lathyrophylla*** | 83.76 | 0.75 | 60.77 | 1.16 | 5.74 |
| ***Corydalis eugeniae*** | 84.17 | 1.00 | 61.26 | 1.72 | 6.60 |
| ***Corydalis caudata*** | 83.47 | 0.97 | 58.64 | 1.13 | 6.77 |
| ***Corydalis decumbens*** | 86.00 | 0.78 | 68.64 | 1.24 | 6.12 |
| ***Corydalis edulis*** | 84.35 | 0.66 | 64.00 | 0.70 | 4.20 |
| ***Corydalis fangshanensis*** | 84.33 | 0.49 | 64.23 | 0.62 | 2.24 |
| ***Corydalis delphinioides*** | 86.29 | 0.61 | 65.40 | 1.11 | 9.00 |
| ***Corydalis kokiana*** | 80.99 | 0.47 | 65.68 | 1.37 | 12.80 |
| **Average** | 83.79 | 0.65 | 64.43 | 1.06 | 6.13 |

Table S4. Comparison of current GC and equilibrium GC (GC*) content between the ITS2 paired and unpaired regions

| Species | ITS2 paired regions | | ITS2 unpaired regions | |
|---|---|---|---|---|
| | GC content (%) | GC* content (%) | GC content (%) | GC* content (%) |
| ***Corydalis yui*** | 85.83 | 73.76 | 62.73 | 62.91 |
| ***Corydalis flexuosa*** | 80.10 | 71.22 | 68.69 | 71.07 |
| ***Corydalis elata*** | 83.87 | 80.72 | 67.86 | 66.54 |
| ***Corydalis benecincta*** | 82.02 | 76.11 | 63.89 | 65.10 |
| ***Corydalis pseudosibirica*** | 86.64 | 74.61 | 58.51 | 60.76 |
| ***Corydalis melanochlora*** | 82.68 | 88.08 | 65.95 | 76.92 |
| ***Corydalis ophiocarpa*** | 82.84 | 72.82 | 64.41 | 64.57 |
| ***Corydalis trachycarpa*** | 82.44 | 76.43 | 63.86 | 67.96 |
| ***Corydalis laucheana*** | 85.03 | 73.23 | 68.48 | 69.88 |
| ***Corydalis incisa*** | 83.73 | 76.40 | 61.81 | 59.85 |
| ***Corydalis zhongdianensis*** | 86.76 | 75.69 | 62.22 | 65.87 |
| ***Corydalis bungeana*** | 85.69 | 73.06 | 66.91 | 63.36 |
| ***Corydalis shensiana*** | 80.87 | 72.83 | 65.65 | 78.79 |
| ***Corydalis rheinbabeniana*** | 83.22 | 76.39 | 62.27 | 71.36 |
| ***Corydalis pachypoda*** | 81.46 | 84.44 | 63.22 | 72.89 |
| ***Corydalis balansae*** | 81.61 | 78.48 | 64.96 | 70.84 |
| ***Corydalis lathyrophylla*** | 83.76 | 78.03 | 60.77 | 59.22 |
| ***Corydalis eugeniae*** | 84.17 | 84.42 | 61.26 | 69.20 |
| ***Corydalis caudata*** | 83.47 | 79.24 | 58.64 | 60.90 |
| **Average** | 83.48 | 77.16 | 63.79 | 67.26 |

Table S5. Comparison of substitution elements from intermediates to GC or AU base-pairs in an initial states of ITS2 rate matrix

| **ITS2 matrix** | **Elements of the ITS2 rate matrix** | | | | | | | | | | | | | | | |
|---|---|---|---|---|---|---|---|---|---|---|---|---|---|---|---|---|
| | GU→GC | CC→GC | GG→GC | GA→GC | UC→GC | AC→GC | NN→GC[a] | $F_{(GC)}$ [a] | GU→AU | AG→AU | AA→AU | CU→AU | UU→AU | AC→AU | NN→AU[a] | $F_{(AU)}$ [a] |
| ***Corydalis yui*** | 2.0814 | 13.8118 | 12.9177 | 12.9177 | 13.8118 | 12.8725 | 68.4129 | 0.45 | 0.282 | 2.6635 | 2.6635 | 1.7504 | 1.7504 | 2.4824 | 11.5922 | 0.0118 |
| ***Corydalis flexuosa*** | 1.9298 | 8.6349 | 7.532 | 7.532 | 8.6349 | 7.4393 | 41.7029 | 0.436 | 0.3848 | 2.5107 | 2.5107 | 1.5018 | 1.5018 | 2.1631 | 10.5729 | 0.0253 |
| ***Corydalis elata*** | 1.3008 | 9.2235 | 8.3515 | 8.3515 | 9.2235 | 5.9733 | 42.4241 | 0.4394 | 0.1972 | 2.1215 | 2.1215 | 1.2662 | 1.2662 | 1.3739 | 8.3465 | 0.0153 |
| ***Corydalis benecincta*** | 2.3459 | 11.7207 | 10.2196 | 10.2196 | 11.7207 | 17.6533 | 63.8798 | 0.4306 | 0.3989 | 3.0742 | 3.0742 | 1.7376 | 1.7376 | 4.6302 | 14.6527 | 0.0192 |
| ***Corydalis pseudosibirica*** | 2.6175 | 14.4411 | 13.4395 | 13.4395 | 14.4411 | 18.6593 | 77.038 | 0.4511 | 0.3134 | 2.6896 | 2.6896 | 1.6091 | 1.6091 | 3.4752 | 12.386 | 0.0101 |
| ***Corydalis melanochlora*** | 1.1091 | 8.107 | 7.1591 | 7.1591 | 8.107 | 4.592 | 36.2333 | 0.434 | 0.1651 | 2.1355 | 2.1355 | 1.0656 | 1.0656 | 1.2096 | 7.7769 | 0.017 |
| ***Corydalis ophiocarpa*** | 0.8815 | 14.7588 | 13.2883 | 13.2883 | 14.7588 | 4.7765 | 61.7522 | 0.441 | 0.1463 | 3.5486 | 3.5486 | 2.2059 | 2.2059 | 1.1485 | 12.8038 | 0.0176 |
| ***Corydalis trachycarpa*** | 1.1079 | 8.4045 | 7.8967 | 7.8967 | 8.4045 | 4.7597 | 38.47 | 0.4331 | 0.1972 | 2.0681 | 2.0681 | 1.4057 | 1.4057 | 1.1712 | 8.316 | 0.019 |
| ***Corydalis sheareri*** | 3.6867 | 0.721 | 0.721 | 0.721 | 0.721 | 0.721 | 7.2917 | 0.3692 | 0.3267 | 0.0639 | 0.0639 | 0.0639 | 0.0639 | 0.0639 | 0.6462 | 0.0327 |
| ***Corydalis curviflora*** | 0.3342 | 0.9581 | 0.9581 | 0.9581 | 0.9581 | 0.9581 | 5.1247 | 0.3531 | 0.045 | 0.129 | 0.129 | 0.129 | 0.129 | 0.129 | 0.69 | 0.0475 |
| ***Corydalis laucheana*** | 1.9557 | 17.7633 | 16.702 | 16.702 | 17.7633 | 11.5451 | 82.4314 | 0.4548 | 0.2975 | 3.5238 | 3.5238 | 2.5405 | 2.5405 | 2.2903 | 14.7164 | 0.0137 |
| ***Corydalis incisa*** | 1.3846 | 11.3371 | 10.3331 | 10.3331 | 11.3371 | 6.4377 | 51.1627 | 0.445 | 0.2065 | 2.6699 | 2.6699 | 1.5412 | 1.5412 | 1.5161 | 10.1448 | 0.0156 |
| ***Corydalis zhongdianensis*** | 2.6438 | 16.2952 | 15.1062 | 15.1062 | 16.2952 | 20.0838 | 85.5304 | 0.4521 | 0.3173 | 2.9824 | 2.9824 | 1.8129 | 1.8129 | 3.6758 | 13.5837 | 0.0099 |
| ***Corydalis bungeana*** | 2.1983 | 19.4145 | 18.3343 | 18.3343 | 19.4145 | 13.7283 | 91.4242 | 0.4579 | 0.3091 | 3.6581 | 3.6581 | 2.578 | 2.578 | 2.5867 | 15.368 | 0.0121 |
| ***Corydalis shensiana*** | 2.0713 | 15.4979 | 13.9021 | 13.9021 | 15.4979 | 13.9391 | 74.8104 | 0.4342 | 0.395 | 4.3312 | 4.3312 | 2.6509 | 2.6509 | 3.8956 | 18.2548 | 0.0231 |
| ***Corydalis rheinbabeniana*** | 1.9509 | 8.218 | 7.6522 | 7.6522 | 8.218 | 7.736 | 41.4273 | 0.4439 | 0.3326 | 1.8959 | 1.8959 | 1.3048 | 1.3048 | 1.7847 | 8.5187 | 0.0175 |
| ***Corydalis pachypoda*** | 2.2414 | 8.3518 | 7.4962 | 7.4962 | 8.3518 | 12.7751 | 46.7125 | 0.4297 | 0.4029 | 2.2523 | 2.2523 | 1.3475 | 1.3475 | 3.4451 | 11.0476 | 0.0208 |
| ***Corydalis balansae*** | 1.1039 | 11.4027 | 10.34 | 10.34 | 11.4027 | 5.2871 | 49.8764 | 0.4356 | 0.2076 | 2.9592 | 2.9592 | 1.9444 | 1.9444 | 1.3721 | 11.3869 | 0.0213 |
| ***Corydalis lathyrophylla*** | 2.0937 | 3.3294 | 3.1 | 3.1 | 3.3294 | 2.2486 | 17.2011 | 0.4382 | 0.3406 | 0.7431 | 0.7431 | 0.5043 | 0.5043 | 0.5019 | 3.3373 | 0.0159 |
| ***Corydalis eugeniae*** | 3.7566 | 2.9367 | 2.764 | 2.764 | 2.9367 | 3.9912 | 19.1492 | 0.4392 | 0.5941 | 0.6327 | 0.6327 | 0.4371 | 0.4371 | 0.8599 | 3.5936 | 0.015 |
| ***Corydalis caudata*** | 0.6925 | 6.6386 | 6.0624 | 6.0624 | 6.6386 | 5.1833 | 31.2778 | 0.3746 | 0.1043 | 1.6035 | 1.6035 | 0.9134 | 0.9134 | 1.252 | 6.3901 | 0.0136 |
| **Average** | 1.8804 | 10.0936 | 9.2512 | 9.2512 | 10.0936 | 8.6362 | 49.2063 | 0.4306 | 0.2840 | 2.2979 | 2.2979 | 1.4433 | 1.4433 | 1.9537 | 9.7202 | 0.0188 |

[a] NN→GC/AU represent the sum substitution rates from the six intermediates to GC or AU; $F_{(GC)}$/ $F_{(AU)}$ represent the GC or AU frequency in ITS2 rate matrix

Table S6. Comparison of substitution elements from intermediates to GC or AU base-pairs at equilibrium states of ITS2 rate matrix

| **ITS2 matrix** | **Elements of the ITS2 rate matrix** | | | | | | | | | | | | | | | |
|---|---|---|---|---|---|---|---|---|---|---|---|---|---|---|---|---|
| | GU→GC | GG→GC | GA→GC | CC→GC | UC→GC | AC→GC | NN→GC[a] | $F_{(GC)}$ [a] | GU→AU | AG→AU | AA→AU | CU→AU | UU→AU | AC→AU | NN→AU[a] | $F_{(AU)}$ [a] |
| ***Corydalis yui*** | 0.5438 | 9.7281 | 9.7281 | 6.6459 | 6.6459 | 2.2024 | 35.4942 | 0.4061 | 0.2468 | 1.4103 | 1.4103 | 4.415 | 4.415 | 0.4674 | 12.3648 | 0.0391 |
| ***Corydalis flexuosa*** | 0.2377 | 1.3756 | 1.3756 | 14.6282 | 14.6282 | 1.3756 | 33.6209 | 0.3827 | 0.1087 | 5.1577 | 5.1577 | 6.4597 | 6.4597 | 0.485 | 23.8285 | 0.0617 |
| ***Corydalis elata*** | 0.2496 | 7.9659 | 7.9659 | 6.0216 | 6.0216 | 0.7533 | 28.9779 | 0.4175 | 0.0501 | 1.7416 | 1.7416 | 1.599 | 1.599 | 0.2179 | 6.9492 | 0.0242 |
| ***Corydalis benecincta*** | 0.5937 | 6.5113 | 6.5113 | 19.3535 | 19.3535 | 8.806 | 61.1293 | 0.3925 | 0.4555 | 3.1203 | 3.1203 | 4.9965 | 4.9965 | 1.4198 | 18.1089 | 0.0486 |
| ***Corydalis pseudosibirica*** | 1.0353 | 6.0897 | 6.0897 | 4.4034 | 4.4034 | 2.1526 | 24.1741 | 0.4022 | 0.1784 | 2.5215 | 2.5215 | 1.0492 | 1.0492 | 1.2327 | 8.5525 | 0.0397 |
| ***Corydalis melanochlora*** | 0.3247 | 1.8528 | 1.8528 | 1.1736 | 1.1736 | 0.4234 | 6.8009 | 0.3771 | 0.04 | 0.1817 | 0.1817 | 0.228 | 0.228 | 0.0656 | 0.925 | 0.0072 |
| ***Corydalis ophiocarpa*** | 0.1642 | 6.9348 | 6.9348 | 6.4989 | 6.4989 | 0.8324 | 27.864 | 0.3787 | 0.0843 | 1.4543 | 1.4543 | 3.5599 | 3.5599 | 0.1863 | 10.299 | 0.0435 |
| ***Corydalis trachycarpa*** | 0.2525 | 4.7227 | 4.7227 | 3.2525 | 3.2525 | 0.7011 | 16.904 | 0.3876 | 0.0912 | 0.7526 | 0.7526 | 1.7062 | 1.7062 | 0.1622 | 5.171 | 0.0324 |
| ***Corydalis sheareri*** | 0.2131 | 5.628 | 5.628 | 5.628 | 5.628 | 5.628 | 28.3531 | 0.3498 | 0.0349 | 0.9206 | 0.9206 | 0.9206 | 0.9206 | 0.9206 | 4.6379 | 0.0572 |
| ***Corydalis curviflora*** | 0.1637 | 5.5714 | 5.5714 | 5.5714 | 5.5714 | 5.5714 | 28.0207 | 0.2991 | 0.0198 | 0.6721 | 0.6721 | 0.6721 | 0.6721 | 0.6721 | 3.3803 | 0.0361 |
| ***Corydalis laucheana*** | 1.3345 | 2.133 | 2.133 | 2.2166 | 2.2166 | 1.3468 | 11.3805 | 0.385 | 0.3383 | 1.0493 | 1.0493 | 0.5407 | 0.5407 | 0.6375 | 4.1558 | 0.0462 |
| ***Corydalis incisa*** | 0.4907 | 3.0076 | 3.0076 | 0.7464 | 0.7464 | 0.5776 | 8.5763 | 0.3629 | 0.1218 | 0.646 | 0.646 | 0.7464 | 0.7464 | 0.2488 | 3.1554 | 0.0388 |
| ***Corydalis zhongdianensis*** | 1.0547 | 6.7744 | 6.7744 | 3.2756 | 3.2756 | 2.1971 | 23.3518 | 0.4014 | 0.3444 | 1.0164 | 1.0164 | 2.2118 | 2.2118 | 0.6817 | 7.4825 | 0.0407 |
| ***Corydalis bungeana*** | 0.752 | 7.2438 | 7.2438 | 7.2332 | 7.2332 | 1.9695 | 31.6755 | 0.4076 | 0.3122 | 2.332 | 2.332 | 3.0071 | 3.0071 | 0.635 | 11.6254 | 0.0546 |
| ***Corydalis shensiana*** | 0.3448 | 3.5918 | 3.5918 | 1.865 | 1.865 | 0.5342 | 11.7926 | 0.3441 | 0.0961 | 1.0341 | 1.0341 | 1.0012 | 1.0012 | 0.2962 | 4.4629 | 0.0532 |
| ***Corydalis rheinbabeniana*** | 0.4214 | 2.8772 | 2.8772 | 1.6714 | 1.6714 | 0.7024 | 10.221 | 0.3681 | 0.1376 | 0.467 | 0.467 | 0.9391 | 0.9391 | 0.1963 | 3.1461 | 0.0336 |
| ***Corydalis pachypoda*** | 0.4799 | 2.9927 | 2.9927 | 1.943 | 1.943 | 0.9082 | 11.2595 | 0.4049 | 0.0998 | 0.2872 | 0.2872 | 0.6222 | 0.6222 | 0.1342 | 2.0528 | 0.0124 |
| ***Corydalis balansae*** | 0.3119 | 4.0553 | 4.0553 | 4.89 | 4.89 | 1.3235 | 19.526 | 0.3954 | 0.11 | 1.023 | 1.023 | 1.4298 | 1.4298 | 0.2769 | 5.2925 | 0.0292 |
| ***Corydalis lathyrophylla*** | 1.0384 | 1.6412 | 1.6412 | 1.6004 | 1.6004 | 1.0126 | 8.5342 | 0.3813 | 0.2673 | 0.4903 | 0.4903 | 0.4225 | 0.4225 | 0.3102 | 2.4031 | 0.0301 |
| ***Corydalis eugeniae*** | 1.458 | 1.8869 | 1.8869 | 1.2379 | 1.2379 | 0.9565 | 8.6641 | 0.3925 | 0.2141 | 0.2995 | 0.2995 | 0.2771 | 0.2771 | 0.2314 | 1.5987 | 0.0139 |
| ***Corydalis caudata*** | 0.329 | 2.9839 | 2.9839 | 1.3437 | 1.3437 | 0.5189 | 9.5031 | 0.3504 | 0.0765 | 0.4398 | 0.4398 | 0.6939 | 0.6939 | 0.1698 | 2.5137 | 0.0267 |
| **Average** | 0.5616 | 4.5509 | 4.5509 | 4.8191 | 4.8191 | 1.9283 | 21.2297 | 0.3803 | 0.1632 | 1.2865 | 1.2865 | 1.7856 | 1.7856 | 0.4594 | 6.7670 | 0.0366 |

[a] NN→GC/AU represent the sum substitution rates from the six intermediates to GC or AU; $F_{(GC)}$/ $F_{(AU)}$ represent the GC or AU frequency in ITS2 rate matrix

Table S7. Mismatches transformation inferring from the best-fit double-substitution rate matrix at initial states of ITS2 rate matrix

| ITS2 matrix | Mismatches transformation | | | | | | | |
|---|---|---|---|---|---|---|---|---|
| | AG | | AC | | GU | | CU | |
| | AG→CG | AG→AU | AC→GC | AC→AU | GU→GC | GU→AU | CU→CG | CU→AU |
| *Corydalis yui* | 12.9177 | 2.6635 | 12.8725 | 2.4824 | 2.0814 | 0.282 | 13.8118 | 1.7504 |
| *Corydalis flexuosa* | 7.532 | 2.5107 | 7.4393 | 2.1631 | 1.9298 | 0.3848 | 8.6349 | 1.5018 |
| *Corydalis elata* | 8.3515 | 2.1215 | 5.9733 | 1.3739 | 1.3008 | 0.1972 | 9.2235 | 1.2662 |
| *Corydalis benecincta* | 10.2196 | 3.0742 | 17.6533 | 4.6302 | 2.3459 | 0.3989 | 11.7207 | 1.7376 |
| *Corydalis pseudosibirica* | 13.4395 | 2.6896 | 18.6593 | 3.4752 | 2.6175 | 0.3134 | 14.4411 | 1.6091 |
| *Corydalis melanochlora* | 7.1591 | 2.1355 | 4.592 | 1.2096 | 1.1091 | 0.1651 | 8.107 | 1.0656 |
| *Corydalis ophiocarpa* | 13.2883 | 3.5486 | 4.7765 | 1.1485 | 0.8815 | 0.1463 | 14.7588 | 2.2059 |
| *Corydalis trachycarpa* | 7.8967 | 2.0681 | 4.7597 | 1.1712 | 1.1079 | 0.1972 | 8.4045 | 1.4057 |
| *Corydalis sheareri* | 0.8578 | 0.0639 | 0.721 | 0.0639 | 3.6867 | 0.3267 | 0.8578 | 0.0639 |
| *Corydalis curviflora* | 1.1792 | 0.129 | 0.9581 | 0.129 | 0.3342 | 0.045 | 1.1792 | 0.129 |
| *Corydalis laucheana* | 16.702 | 3.5238 | 11.5451 | 2.2903 | 1.9557 | 0.2975 | 17.7633 | 2.5405 |
| *Corydalis incisa* | 10.3331 | 2.6699 | 6.4377 | 1.5161 | 1.3846 | 0.2065 | 11.3371 | 1.5412 |
| *Corydalis zhongdianensis* | 15.1062 | 2.9824 | 20.0838 | 3.6758 | 2.6438 | 0.3173 | 16.2952 | 1.8129 |
| *Corydalis bungeana* | 18.3343 | 3.6581 | 13.7283 | 2.5867 | 2.1983 | 0.3091 | 19.4145 | 2.578 |
| *Corydalis shensiana* | 13.9021 | 4.3312 | 13.9391 | 3.8956 | 2.0713 | 0.395 | 15.4979 | 2.6509 |
| *Corydalis rheinbabeniana* | 7.6522 | 1.8959 | 7.736 | 1.7847 | 1.9509 | 0.3326 | 8.218 | 1.3048 |
| *Corydalis pachypoda* | 7.4962 | 2.2523 | 12.7751 | 3.4451 | 2.2414 | 0.4029 | 8.3518 | 1.3475 |
| *Corydalis balansae* | 10.34 | 2.9592 | 5.2871 | 1.3721 | 1.1039 | 0.2076 | 11.4027 | 1.9444 |
| *Corydalis lathyrophylla* | 3.1 | 0.7431 | 2.2486 | 0.5019 | 2.0937 | 0.3406 | 3.3294 | 0.5043 |
| *Corydalis eugeniae* | 2.764 | 0.6327 | 3.9912 | 0.8599 | 3.7566 | 0.5941 | 2.9367 | 0.4371 |
| *Corydalis caudata* | 6.0624 | 1.6035 | 5.1833 | 1.252 | 0.6925 | 0.1043 | 6.6386 | 0.9134 |
| **Average** | 9.2683 | 2.2979 | 8.6362 | 1.9537 | 1.8804 | 0.2840 | 10.1107 | 1.4433 |

Table S8. Mismatches transformation inferring from the best-fit double-substitution rate matrix at equilibrium states ITS2 rate matrix

| **ITS2 matrix** | **Mismatches transformation** | | | | | | | |
|---|---|---|---|---|---|---|---|---|
| | **AG** | | AC | | **GU** | | CU | |
| | **AG→CG** | **AG→AU** | AC→GC | AC→AU | **GU→GC** | **GU→AU** | CU→CG | CU→AU |
| ***Corydalis yui*** | 9.7281 | 1.4103 | 2.2024 | 0.4674 | 0.5438 | 0.2468 | 6.6459 | 4.415 |
| ***Corydalis flexuosa*** | 14.1245 | 5.1577 | 1.3756 | 0.485 | 0.2377 | 0.1087 | 14.6282 | 6.4597 |
| ***Corydalis elata*** | 7.9659 | 1.7416 | 0.7533 | 0.2179 | 0.2496 | 0.0501 | 6.0216 | 1.599 |
| ***Corydalis benecincta*** | 6.5113 | 3.1203 | 8.806 | 1.4198 | 0.5937 | 0.4555 | 19.3535 | 4.9965 |
| ***Corydalis pseudosibirica*** | 6.0897 | 2.5215 | 2.1526 | 1.2327 | 1.0353 | 0.1784 | 4.4034 | 1.0492 |
| ***Corydalis melanochlora*** | 1.8528 | 0.1817 | 0.4234 | 0.0656 | 0.3247 | 0.04 | 1.1736 | 0.228 |
| ***Corydalis ophiocarpa*** | 6.9348 | 1.4543 | 0.8324 | 0.1863 | 0.1642 | 0.0843 | 6.4989 | 3.5599 |
| ***Corydalis trachycarpa*** | 4.7227 | 0.7526 | 0.7011 | 0.1622 | 0.2525 | 0.0912 | 3.2525 | 1.7062 |
| ***Corydalis sheareri*** | 5.7499 | 0.9206 | 5.628 | 0.9206 | 0.2131 | 0.0349 | 5.7499 | 0.9206 |
| ***Corydalis curviflora*** | 8.0881 | 0.6721 | 5.5714 | 0.6721 | 0.1637 | 0.0198 | 8.0881 | 0.6721 |
| ***Corydalis laucheana*** | 2.133 | 1.0493 | 1.3468 | 0.6375 | 1.3345 | 0.3383 | 2.2166 | 0.5407 |
| ***Corydalis incisa*** | 3.0076 | 0.646 | 0.5776 | 0.2488 | 0.4907 | 0.1218 | 1.4999 | 0.7464 |
| ***Corydalis zhongdianensis*** | 6.7744 | 1.0164 | 2.1971 | 0.6817 | 1.0547 | 0.3444 | 3.2756 | 2.2118 |
| ***Corydalis bungeana*** | 7.2438 | 2.332 | 1.9695 | 0.635 | 0.752 | 0.3122 | 7.2332 | 3.0071 |
| ***Corydalis shensiana*** | 3.5918 | 1.0341 | 0.5342 | 0.2962 | 0.3448 | 0.0961 | 1.865 | 1.0012 |
| ***Corydalis rheinbabeniana*** | 2.8772 | 0.467 | 0.7024 | 0.1963 | 0.4214 | 0.1376 | 1.6714 | 0.9391 |
| ***Corydalis pachypoda*** | 2.9927 | 0.2872 | 0.9082 | 0.1342 | 0.4799 | 0.0998 | 1.943 | 0.6222 |
| ***Corydalis balansae*** | 4.0553 | 1.023 | 1.3235 | 0.2769 | 0.3119 | 0.11 | 4.89 | 1.4298 |
| ***Corydalis lathyrophylla*** | 1.6412 | 0.4903 | 1.0126 | 0.3102 | 1.0384 | 0.2673 | 1.6004 | 0.4225 |
| ***Corydalis eugeniae*** | 1.8869 | 0.2995 | 0.9565 | 0.2314 | 1.458 | 0.2141 | 1.2379 | 0.2771 |
| ***Corydalis caudata*** | 2.9839 | 0.4398 | 0.5189 | 0.1698 | 0.329 | 0.0765 | 1.3437 | 0.6939 |
| **Average** | 5.2836 | 1.2865 | 1.9283 | 0.4594 | 0.5616 | 0.1632 | 4.9806 | 1.7856 |

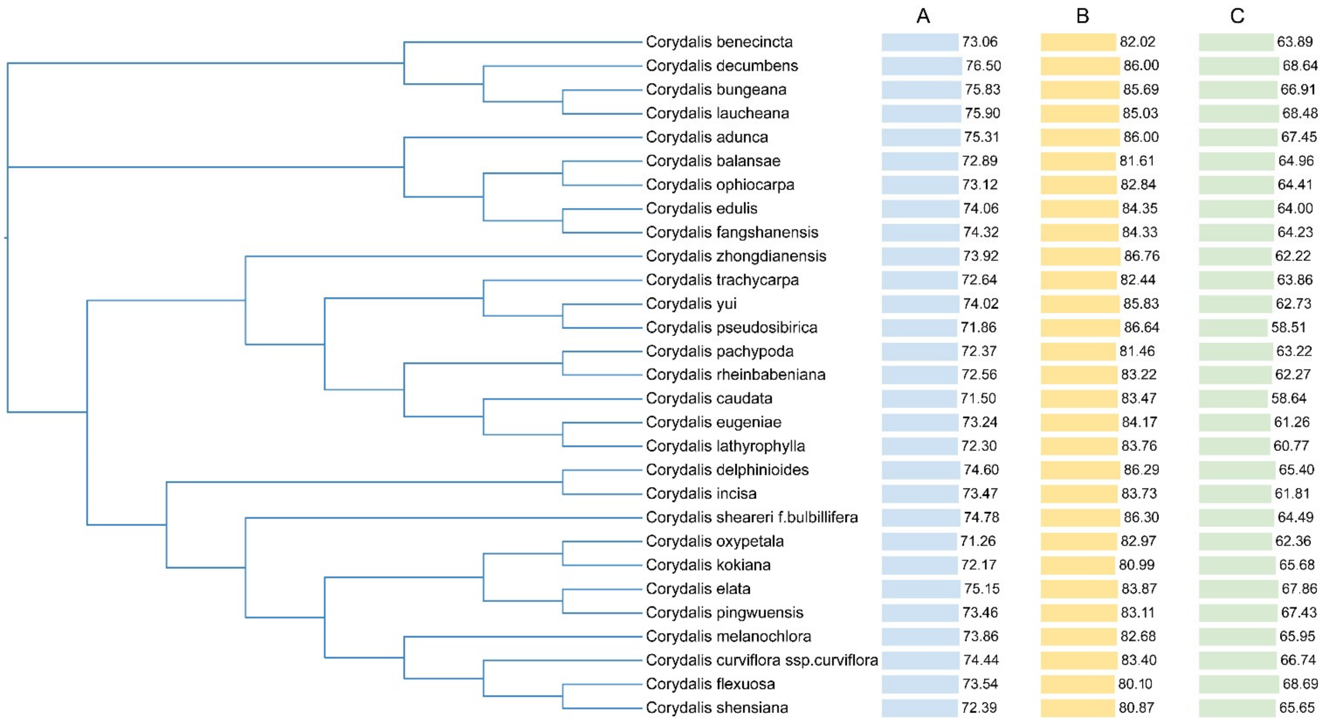


**Figure S1.** Variation of ITS2 GC content across the 29 *Corydalis* phylogeny. A, B and C value followed species name represents GC content of the entire ITS2 region, ITS2 paired region and ITS2 unpaired region, respectively. The phylogeny was constructed from based on the recent phylogeny of *Corydalis* (Xu et al., 2022).
